# Unsupervised cluster analysis of patients with recovered left ventricular ejection fraction identifies unique clinical phenotypes

**DOI:** 10.1371/journal.pone.0248317

**Published:** 2021-03-18

**Authors:** Andrew Perry, Francis Loh, Luigi Adamo, Kathleen W. Zhang, Elena Deych, Randi Foraker, Douglas L. Mann

**Affiliations:** 1 Cardiovascular Division, Department of Medicine, Washington University School of Medicine, St. Louis, Missouri, United States of America; 2 Institute for Informatics, Washington University School of Medicine, St. Louis, Missouri, United States of America; King’s College London, UNITED KINGDOM

## Abstract

**Background:**

Patients with heart failure (HF) with recovered ejection fraction (HFrecEF) are a recently identified cohort that are phenotypically and biologically different from HFrEF and HFpEF patients. Whether there are unique phenotypes among HFrecEF patients is not known.

**Methods:**

We studied all patients at a large medical center, who had an improvement in LVEF from ≤ 35% to ≥ 50% (LVrecEF) between January 1, 2005 and December 31, 2013. We identified a set of 11 clinical variables and then performed unsupervised clustering analyses to identify unique clinical phenotypes among patients with LVrecEF, followed by a Kaplan-Meier analysis to identify differences in survival and the proportion of LVrecEF patients who maintained an LVEF ≥ 50% during the study period.

**Results:**

We identified 889 patients with LVrecEF who clustered into 7 unique phenotypes ranging in size from 37 to 420 patients. Kaplan-Meier analysis demonstrated significant differences in mortality across clusters (logrank p<0.0001), with survival ranging from 14% to 87% at 1000 days, as well as significant differences in the proportion of LVrecEF patients who maintained an LVEF ≥ 50%.

**Conclusion:**

There is significant clinical heterogeneity among patients with LVrecEF. Clinical outcomes are distinct across phenotype clusters as defined by clinical cardiac characteristics and co-morbidities. Clustering algorithms may identify patients who are at high risk for recurrent HF, and thus be useful for guiding treatment strategies for patients with LVrecEF.

## Introduction

Heart failure (HF) with a recovered left ventricular ejection fraction (HFrecEF) refers to a recently identified sub-group of HF patients with a reduced ejection fraction (HFrEF) whose left ventricular (LV) ejection fraction (LVEF) improves in response to implementation of guideline directed medical therapy (GDMT) or device therapy [1]. Importantly, the subgroup of HFrecEF patients are clinically distinct from patients with HF with a preserved ejection fraction (HFpEF), who also have an LVEF > 50% along with the presence of HF signs and symptoms [2, 3].

Although recovery of LV function is associated with improved clinical outcomes in HFrecEF patients when compared to HFrEF, there is a growing body of evidence suggesting that even among patients who experience a complete normalization of LV structure and function after implementation of GDMT, a significant proportion will develop recurrent LV dysfunction accompanied by recurrent HF events [3, 4]. The biological explanation for why some patients have improved LVEF remain free from HF events indefinitely (“myocardial recovery”) and others who have a similar improvement in LVEF stabilize initially, but continue to experience recurrent HF events (“myocardial remission”) is not known, and represents a significant knowledge gap [5].

Machine learning algorithms offer novel ways to explore relationships which might not be readily apparent. In contrast to supervised learning algorithms which can be used to learn about relationships of input variables to a fixed set of outcomes, unsupervised learning algorithms attempt to identify naturally occurring patterns or groupings within the data set without information regarding any particular outcome [6]. Given the complexity and heterogeneity of HFrecEF patients, we sought to use unsupervised machine learning to identify unique subsets (clusters) of patients with recovered LVEF (LVrecEF), with the goal of identifying low- and high-risk subsets of LVrecEF patients, analogous to the approach that was taken to identify clinical phenotypes in patients with HFpEF [7]. Here we show that there is a previously unrecognized heterogeneity of clinical phenotypes for patients with LVrecEF, and that the clinical outcomes of these different phenotypes differ depending on patient characteristics and co-morbidities.

## Methods

We conducted a retrospective cohort study of all patients at Barnes-Jewish Hospital who had a depressed LVEF (≤ 35%) on a 2-D echocardiogram, with recovery of LVEF ≥ 50% on a subsequent 2-D echocardiogram that was obtained for routine clinical reasons [1]. Since the LVEF data was used from the clinical echocardiogram reports with errors as high as 10%, we chose the threshold of 35% rather than 40% as is typically used to ensure our study population had truly reduced LVEF [8]. We obtained 2-D echocardiographic data between January 1, 2005 and December 31, 2013 and collected mortality data from the Social Security Death Index, which was available through December 31, 2014. Echocardiographic data was obtained from the clinical echocardiogram report. We pre-specified that our cohort would exclude those with recovery of LVEF following cardiac transplantation or following placement of a left ventricular assist device (LVAD). Demographic data were obtained from Barnes-Jewish Hospital and Washington University electronic medical records. We obtained data on the QRS duration from the computerized measurements of ECGs performed within 6 months of the “recovery” 2-D echocardiogram, and we defined minority as any race other than “white” or “Caucasian”. Co-morbidities were assessed by ICD-9 and ICD-10 codes for inpatient and outpatient visits using the Elixhauser co-morbidity index [9] (details shown in Table 1).

**Table 1:** ICD and CPT codes used to define co-morbidities.

Co-morbidity	ICD/CPT Codes
Ischemic heart disease	410.70, 410.90, 412.00, 414.01, 414.02, 414.05, 414.80, 414.90, I25.2, I25.5 I25.810, V45.81, Z95.1
Atrial Fibrillation	427.31, I48.0, I48.1, I48.2, I48.91
Diabetes	250.00–250.33, 250.40–250.73, 250.90–250.93
Cardiac Resynchronization Therapy	V53.32

We obtained medication data from the outpatient electronic medical record. Active medications were defined as being prescribed or renewed within the 6 month period prior or after the time that LVEF recovery was documented. This study was approved by the Washington University Institutional Review Board waived the need for informed consent.

### Clinical phenotyping of LVrecEF patients

We prospectively identified a set of 11 clinical variables that were previously shown to predict clinical outcomes in patients with HFrEF or HFrecEF: age, weight, LVEF, history of atrial fibrillation, history of diabetes, ischemic heart disease, cardiac resynchronization therapy (CRT), moderate to severe mitral regurgitation, QRS ≥120ms, and time to LVEF recovery [1, 5, 10–17]. We used an unsupervised clustering algorithm termed partition around the medoids (PAM), to partition patients into different clinical clusters based on the differences in the pre-specified set of 11 clinical variables.

The PAM algorithm partitions a set of variables into two or more clusters by finding a set of representative objects called medoids, such that data points within a cluster are similar and data points in different clusters are dissimilar. The medoid of a cluster is defined as that data point for which the average dissimilarity to all other data points in the cluster is minimal, i.e., the most centrally-located point in the cluster. Partitioning of clusters was performed using the “pam” function from the “cluster” package in R (v3.6.1, 2019). Gower distance was used as the distance metric, as our clinical data consisted of mixed data types. We determined the optimal number of clusters using the silhouette width method. All clustering was performed in a blinded manner with respect to patient outcomes, which were determined after defining the clusters.

### Statistical methods

The primary outcome was all-cause mortality among different clusters of patients with LVEF recovery, determined by Kaplan-Meier analysis using a log-rank test. The secondary outcome was the proportion of patients within a cluster who maintained an LVEF ≥ 50% during follow-up, determined by Kaplan-Meier analysis using a log-rank test. We defined “baseline” as being the date of the echocardiogram with the first recorded LVEF ≥ 50%. When the overall difference among the clusters was significant, the differences between individual clusters by pair-wise comparisons were assessed using a Bonferroni multiple-comparison adjustment. We analyzed differences in the use of evidence-based medication use among the different clusters by Chi-squared test or Fisher’s exact test, as appropriate, and the effect of loss of recovery on survival as a time-dependent covariate in a Cox proportional hazard model. Statistical analyses for creation of phenotypic groups were performed using the statistical programming language R (v3.6.1).

## Results

### Study population

**Fig 1:**
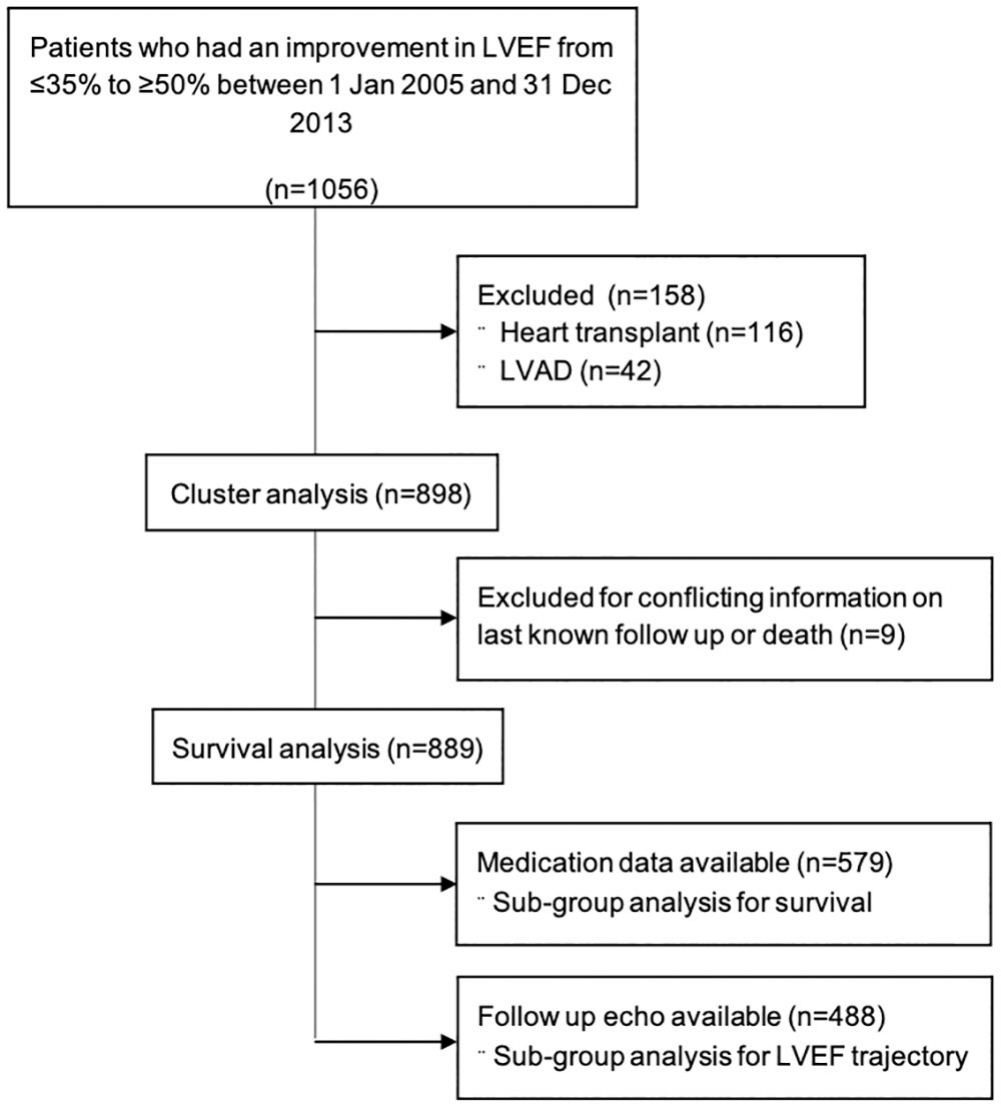
Consort diagram of patients included in the final analysis. LVEF = left ventricular ejection fraction. LVAD = left ventricular assist device.

We identified 1,056 consecutive patients who had an increase in their LVEF from ≤35% to ≥ 50% during the specified study period. As shown in the consort diagram (Fig 1), we excluded 116 patients who had heart transplantation and 42 patients who had an LVAD implanted. After creation of the clusters, when comparing outcomes, 9 patients were excluded because of conflicting data regarding last known follow-up or death. The final patient cohort consisted of 889 patients with LVrecEF, of which ~ 13% (119 of 889 patients) had ischemic heart disease.

### Cluster formation

The silhouette analysis suggested that the optimal number of clusters was 7 (S1 Fig). For simplicity, we arbitrarily numbered the clusters based on probability of survival, with cluster 1 having the greatest mortality and cluster 7 having the lowest mortality (Fig 1). The demographic features of each of the clusters are shown in Table 2. Sizes of clusters ranged from n = 37 to n = 420 and were distinguished by the type and frequency of co-morbidities within each cluster. All of the patients in Cluster 1 had a QRS duration ≥ 120ms, of whom only 3 of 37 had CRT. Cluster 2 comprised patients who all had a diagnosis of diabetes mellitus. Cluster 3 comprised patients without any of the pre-specified co-morbidities or CRT. Cluster 4 comprised patients who had CRT. Meanwhile, Cluster 5 comprised patients who had a diagnosis of atrial fibrillation, Cluster 6 comprised patients who had both atrial fibrillation and CRT, and Cluster 7 comprised of patients who had diagnoses associated with ischemic heart disease.

**Table 2:** Characteristics of patients by cluster at time of LVEF recovery.

Cluster	All	1	2	3	4	5	6	7
**Number of patients**	889	37	87	420	88	123	64	70
**Age (median [IQR])**	61 [51, 71]	69 [59, 84]	56 [49, 67]	60 [48, 70]	57 [49, 68]	67 [57, 78]	65 [56, 78]	63 [56, 73]
**BMI (median [IQR])**	24 [20, 28]	21.2 [18.1, 26.0]	24.0 [20.6, 33.1]	23.6 [19.8, 27.6]	24.4 [20.6, 28.6]	24.2 [21.5, 29.0]	24.4 [21.1, 27.3]	24.6 [21.7, 28.8]
**Female (%)**	415 (46.7)	17 (45.9)	37 (42.5)	227 (54.2)	46 (52.3)	43 (35.0)	23 (35.9)	22 (31.4)
**Minority (%)**	319 (35.9)	11 (29.7)	48 (55.2)	157 (37.4)	32 (36.4)	30 (24.4)	18 (28.1)	23 (32.9)
**Hypertension**	707 (79.5)	31 (83.8%)	74 (85.1%)	294 (70.0%)	74 (84.1%)	107 (87.0%)	68 (97.1%)	59 (92.2%)
**Systolic blood pressure (median [IQR])**	122 [106, 139]	117 [93, 128]	128 [107, 145]	121 [105, 139]	124 [104, 137]	122 [110, 139]	122 [116, 130]	121 [108, 140]
**Atrial fibrillation (%)**	206 (23.2)	5 (13.5)	7 (8.0)	0 (0.0)	0 (0.0)	123 (100.0)	64 (100.0)	7 (10.0)
**Diabetes (%)**	131 (14.7)	3 (8.1)	87 (100.0)	0 (0.0)	15 (17.0)	7 (5.7)	11 (17.2)	8 (11.4)
**Heart rate (median [IQR])**	79 [69, 90]	80 [69, 93]	82 [71, 93]	80 [71, 92]	77 [65, 90]	77 [66, 87]	76 [60,83]	74 [68,82]
**Hemoglobin, mg/dL (median [IQR])**	11.0 [9.5, 12.7]	10.8 [9.1, 12.0]	9.8 [8.6, 11.8]	10.6 [9.5, 12.3]	12.0 [9.9, 13.1]	12.0 [9.9, 13.6]	11.5 [10.1, 13.1]	12.2 [11.1, 13.5]
**Sodium mmol/L (median [IQR])**	139 [137, 142]	139 [136, 142]	139 [137, 141]	140 [137, 142]	140 [137, 142]	139 [137, 142]	139 [137, 142]	140 [138, 142]
**Creatinine (median [IQR])**	1.10 [0.81, 1.63]	1.27 [0.81, 2.04]	1.42 [0.92, 2.80]	1.01 [0.75, 1.50]	1.10 [0.86, 1.36]	1.19 [0.94, 1.51]	1.17 [0.90, 1.66]	1.12 [0.90, 1.42]
**Estimated GFR (median [IQR])**	66 [39, 91]	51 [31, 87]	51 [20, 85]	70 [42, 95]	72 [47, 90]	62 [41, 81]	63 [40, 79]	66 [49, 83]
**Ischemic heart disease (%)**	119 (13.4)	0 (0.0)	7 (8.0)	0 (0.0)	12 (13.6)	9 (7.3)	21 (32.8)	70 (100.0)
**Cardiac resynchronization therapy (%)**	172 (19.3)	3 (8.1)	5 (5.7)	0 (0.0)	88 (100.0)	0 (0.0)	64 (100.0)	12 (17.1)
**Time to recovery, days (median [IQR])**	507 [112, 1115]	237 [52, 655]	484 [131, 980]	241 [35, 773]	938 [465, 1644]	714 [241, 1385]	1095 [759, 1759]	804 [327, 1399]
**Pre-recovery LVEF (median [IQR])**	28 [20, 33]	29 [22, 33]	30 [21, 34]	28 [22, 33]	23 [17, 29]	28 [23, 33]	26 [20, 30]	30 [22, 33]
**Recovered LVEF (median [IQR])**	59 [54, 63]	63 [54, 63]	62.50 [55, 63]	59 [55, 63]	50 [50, 63]	58 [55, 63]	55 [52, 63]	59 [53, 63]
**Change in LVEF (median [IQR])**	29 [22, 37]	28 [21, 38]	30 [25, 37]	30 [23, 38]	29 [20, 36]	28 [22, 35]	29 [21, 34]	28 [20, 37]
**Mod-Severe MR (%)**	49 (5.5)	4 (10.8)	3 (3.4)	19 (4.5)	5 (5.7)	6 (4.9)	6 (9.4)	6 (8.6)
**QRS≥120ms (%)**	49 (5.5)	37 (100.0)	3 (3.4)	0 (0.0)	2 (2.3)	2 (1.6)	4 (6.2)	1 (1.4)
**ACEi/ARB (%)**	320 (55.9)	4 (22.2)	26 (49.1)	126 (58.3)	47 (65.3)	55 (55.6)	29 (50.9)	33 (57.9)
**Beta-blocker (%)**	390 (68.2)	11 (61.1)	36 (67.9)	144 (66.7)	52 (72.2)	63 (63.6)	39 (68.4)	45 (78.9)
**MRA (%)**	137 (24.0)	2 (11.1)	10 (18.9)	50 (23.1)	28 (38.9)	18 (18.2)	18 (31.6)	11 (19.3)
**Loop diuretic (%)**	238 (41.6)	10 (55.6)	25 (47.2)	85 (39.4)	37 (51.4)	48 (48.5)	22 (38.6)	11 (19.3)

IQR = interquartile range; BMI = body mass index; GFR = glomerular filtration rate; LVEF = left ventricle ejection fraction; MR = mitral regurgitation; ACEi = angiotensin converting enzyme inhibitor; ARB = angiotensin II receptor blocker; MRA = mineralocorticoid receptor antagonist.

Kaplan-Meier survival analysis of the different clusters showed marked differences in survival at 1000 days. Log-rank analysis showed that the overall differences in mortality among LVrecEF Clusters 1–7 was statistically significant (P <0.0001); however, the only cluster with a statistically different mortality in post-hoc analysis adjusted for multiple comparisons was Cluster 1 (p<0.001).

### Use of guideline directed medical therapy

**Fig 2:**
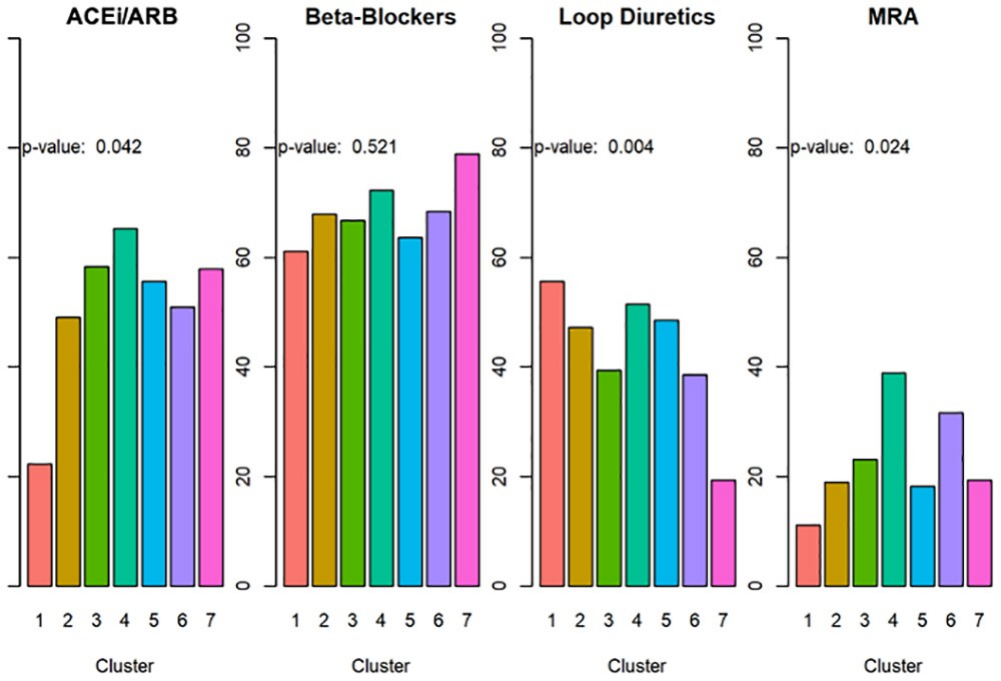
Medication use across cluster. Medication data was available in 579 of 889 patients (65%). Cluster 1 had significantly less use of ACEi/ARB and MRA. There was no significant difference in beta-blocker use among the patient Clusters.

Given that guideline directed medical therapy (GDMT) is associated with clinical stability in HFrecEF patients [1], we examined medication use within each cluster. Medication data were available for 579 of 889 patients (65%). Table 3 displays the proportion of patients in each cluster for whom medication data were available, whereas Fig 2 shows the proportion of patients in each cluster who were receiving ACEi/ARB, beta-blockers, MRAs, and loop diuretics. As shown in Fig 2, there were significant differences in medication use among clusters 1–7 including use of loop diuretics (p = 0.004), MRA (p = 0.024), and ACEi/ARB (p = 0.042); there were, however, no significant differences (p = 0.521) in the use of beta-blockers across clusters.

**Table 3:** Summary of medication data and mortality within patient clusters.

Cluster	Number of patients with medication data (% total patients in cluster)	Number of deaths in patients with medication data (% deaths in cluster)
**Cluster 1**	18 (47)	17 (94)
**Cluster 2**	53 (61)	18 (34)
**Cluster 3**	218 (52)	57 (26)
**Cluster 4**	73 (82)	15 (21)
**Cluster 5**	102 (81)	23 (23)
**Cluster 6**	57 (81)	11 (19)
**Cluster 7**	58 (89)	9 (16)

We examined patient mortality among clusters 1–7 for the patients whose medication data were available (Table 3). This sensitivity analysis showed that mortality was highest in Cluster 1 (94%) and lowest in Cluster 7 (16%), which is similar to the rank ordering for the entire patient cohort by Kaplan-Meier analysis. While there was a statistically significant difference in mortality among the clusters (Chi-squared test, p = 0.005); the only cluster that had a distinct mortality profile after adjusting from multiple comparisons was Cluster 1 (p = 0.0005). Given the small numbers of patients on different medical therapies in each of the different LVrecEF clusters, it was not possible to perform an adjusted regression model to determine whether differences in medication use contributed to differences in mortality among clusters 1–7. For example, there was only 1 survivor in cluster 1, who was not receiving any GDMT for HF.

### Maintenance of LVEF ≥ 50%

**Fig 3:**
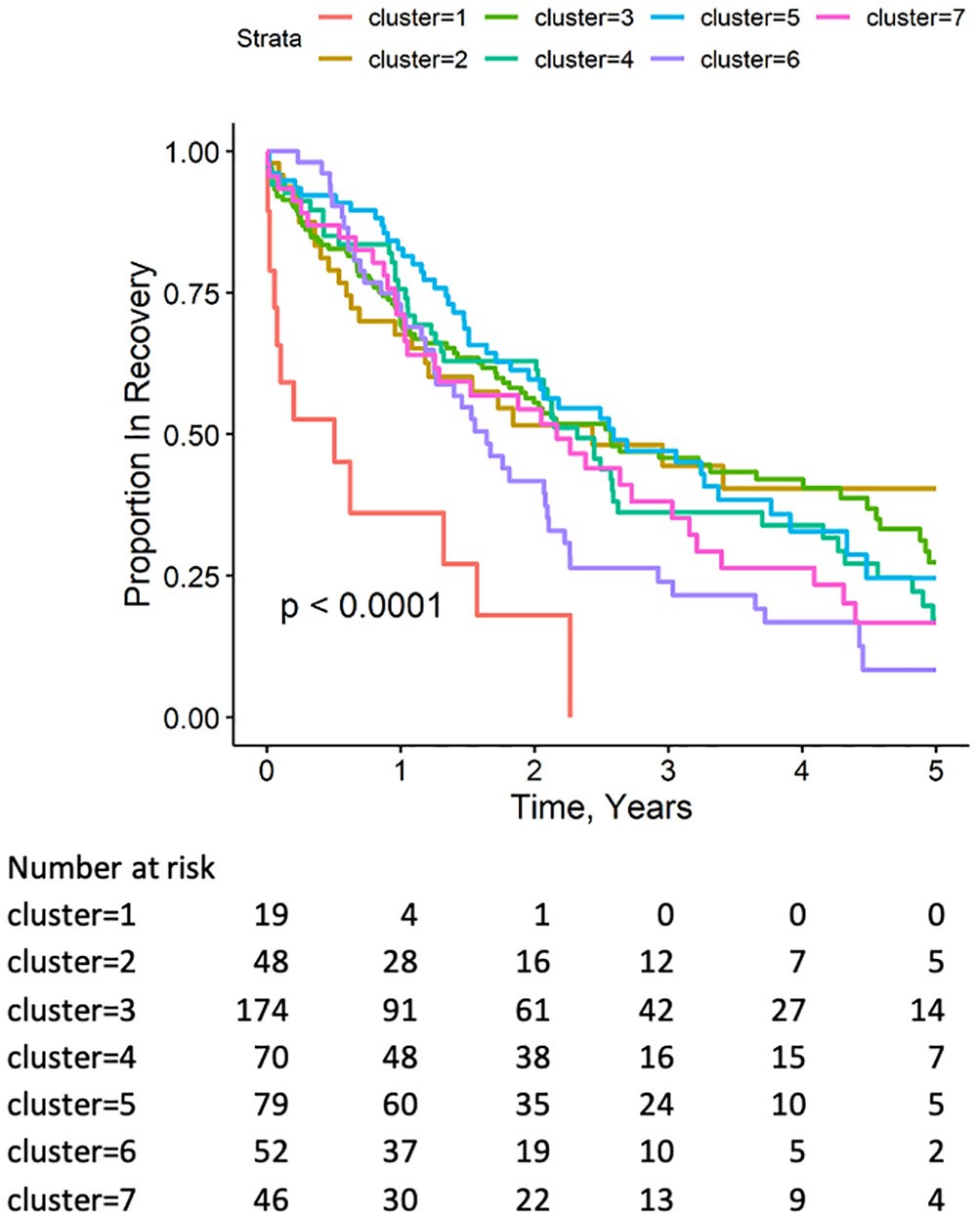
Probability of maintaining LVEF ≥ 50% during follow up. Follow up echo was available in 488/889 patients. Patients in Cluster 1 were the most likely to not have a sustained LVEF ≥ 50% during follow up. Clusters 2–7 had similarly high likelihoods of having LVEF ≥ 50% during follow up.

Prior studies have shown that deterioration of LVEF is associated with worse clinical outcomes in HFrecEF patients [3, 4]. Given the observed differences in morality among the different clusters, we determined the number of patients within each cluster who maintained an LVEF ≥ 50% throughout. Follow-up 2-D echocardiograms were available in 488 of the 889 patients who recovered their LVEF. Patients with a follow up echocardiogram were more likely to have atrial fibrillation, diabetes, ischemic heart disease, CRT, a longer time to recovery, and a lower baseline LVEF (S1 Table). Fig 3 shows two important findings with respect to the proportion of patients within each cluster who maintained LVEF ≥ 50% during follow up. First, there was a significant (p < 0.0001) overall decrease in the proportion of patients who maintained an LVEF ≥ 50% over time. The proportion of LVrecEF patients with an LVEF ≥ 50% decreased during the first year of follow-up and continued to decrease over the ensuing 1–2 years, regardless of the LVrecEF phenotype. The second important finding is that Cluster 1 had the greatest proportion of patients with a decrease in LVEF to ≥ 50% and was significantly different (p<0.01), from all of the other clusters. There were no significant differences in the proportion of patients who maintained an LVEF ≥ 50% in clusters 2–7.

**Fig 4:**
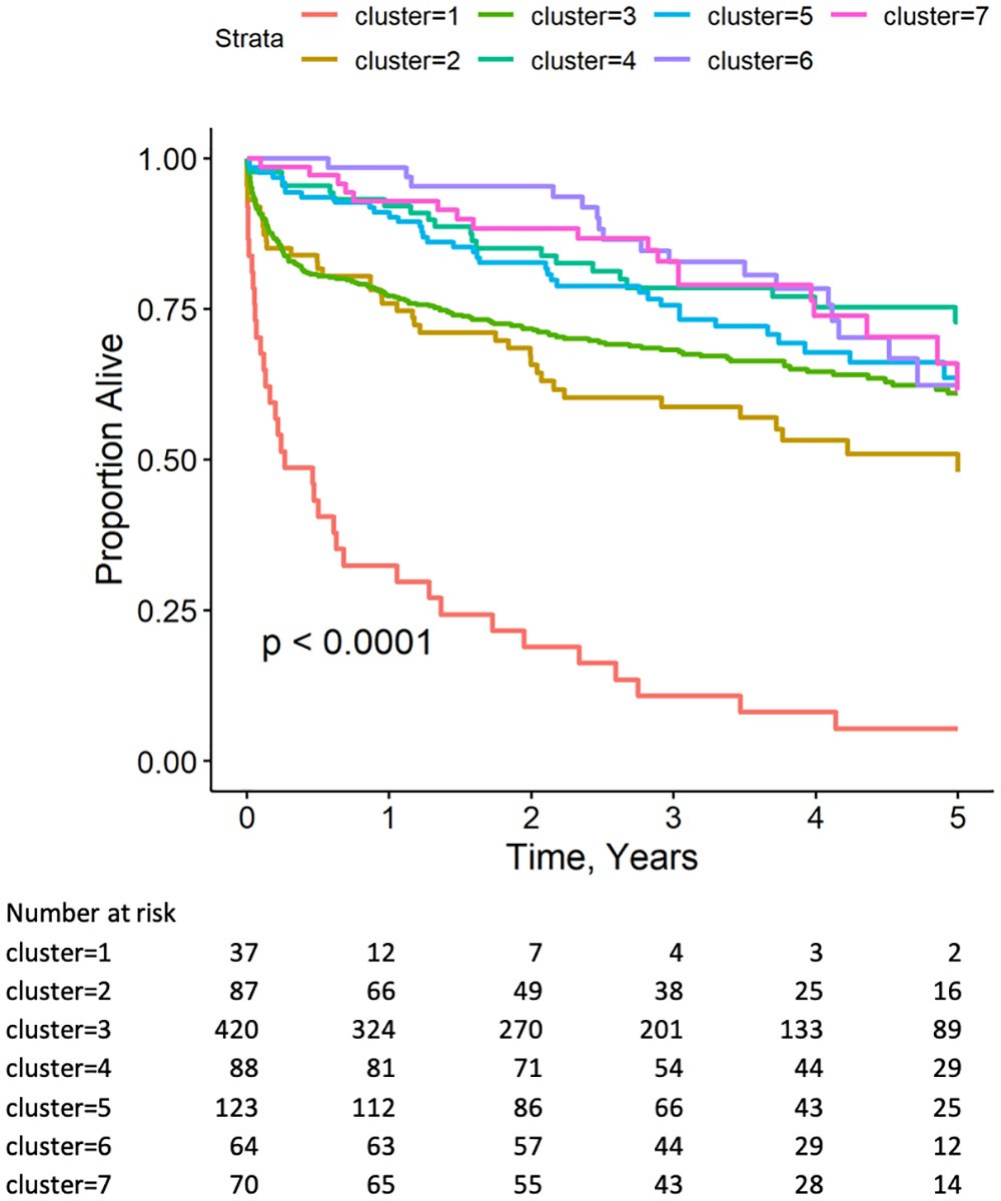
Kaplan-Meier analysis of mortality across clusters. There are significant differences in mortality across phenotype clusters with cluster 1 having the highest mortality, Log-rank p<0.0001. Time to death was measured from the time of LVEF recovery.

In a Cox proportional hazard model, loss of a preserved LVEF was associated with increased mortality (HR, 95% CI: 4.87, 3.44–6.89, p-value < 0.0001). It is notable that with the exception of Cluster 1, where the mortality curves (Fig 4) and the curves depicting the proportion of patients with an LVEF ≥ 50% (Fig 3) were similar, the curves depicting the proportion of patients in each cluster who maintained and LVEF ≥ 50% were not necessarily concordant with changes in mortality determined by Kaplan-Meier analysis.

## Discussion

We used an unsupervised clustering algorithm to identify unique clinical phenotypes among patients with LVrecEF using a set of limited clinical parameters that are readily available within electronic medical records. The major new findings of this study are (1) there is significant clinical heterogeneity among LVrecEF patients and (2) LVrecEF patients span a range of risk with respect to clinical outcomes depending on patient characteristics and co-morbidities. Viewed together, these findings reveal a previously unrecognized heterogeneity of clinical phenotypes and outcomes for LVrecEF patients, which may have important clinical ramifications for how these patients are managed clinically.

### Natural history of recovery of LV ejection fraction in HFrEF patients

A growing body of evidence suggests that even among HFrecEF patients who experience a complete normalization of LVEF, a significant proportion will develop recurrent LV dysfunction accompanied by recurrent HF events [1, 5]. Prior reviews on this topic have emphasized that the great majority of clinical examples of spontaneous recovery of LVEF that occur following transient myocardial injury (e.g., energetic defects or myocardial toxins) are associated with sustained clinical stability, even when the LVEF is severely depressed. In contrast recovery of LVEF is less durable following long-standing and/or permanent injury (e.g., myocardial infarction, genetic abnormalities) [18, 19], suggesting that the extent of myocardial end-organ damage is one plausible explanation for the recurrence of HF in HFrecEF patients [5]. Indeed, we have shown previously that HFrecEF patients who have normalization of LVEF > 50%, but who have a reduced LV global longitudinal strain < 16%, were more likely to experience recurrence of LV dysfunction [20]. Our prior observations with respect to the role of LV global longitudinal strain are consistent with the observations in the present study that save for Cluster 1, wherein the deterioration of LVEF was associated with worsening clinical outcomes (Figs 3 and 4), there was no consistent relationship between clinical outcomes and preservation of an LVEF > 50%. This suggests that assessment of LVEF alone may be insensitive for detecting subsequent events in HFrecEF patients, and that advanced imaging modalities and biomarkers may have additive value for detecting future clinical deterioration.

At the time of this writing, there are limited studies that have evaluated the outcomes of HFrecEF patients. Basuray and colleagues showed that HFrecEF patients had persistent biomarker evidence of inflammation, neurohormonal activation, and myocardial injury, as well as a different clinical course than patients with HFrEF and HFpEF [3]. In their study, HFrecEF patients were the least likely to die, undergo LVAD implantation, or undergo heart transplantation; however, the mortality rate at 8 years in HFrecEF patients was still quite high at 20%. Moreover, HFrecEF patients had a risk of HF hospitalizations that was similar to that of HFpEF patients (HR 1.3 (95% confidence interval, 0.90–2.0; *P* = 0.15)). Similar findings were reported by Lupon et al, who observed that patients with HFrecEF had significantly lower all-cause, cardiovascular, heart failure related and sudden death relative to HFrEF patients [21].

In another outcomes study from the Heart Muscle Disease Registry of Trieste, Merlo and colleagues reported that 63 out of 408 (15%) of dilated cardiomyopathy patients recovered their LVEF > 50% and normalized their LV end-diastolic dimension on GDMT. Importantly, approximately 30% of this subgroup experienced a subsequent decline in LVEF, and 19% required heart transplant or died after 15 years of follow up [4]. Lupon et al. showed that in long term measurements of LVEF HFrEF patients were characterized by an inverted “U” shape, with a marked rise in LVEF during the first year of GDMT implementation, followed by a slow decline in LVEF over the ensuing decade. The authors reported that this pattern was more pronounced in non-ischemic HF and in women, consistent with prior observations that suggest female gender, non-ischemic etiology, younger age, absence of left bundle branch block (LBBB) and a shorter duration of HF are associated with reverse LV remodeling and recovery of LVEF [1]. Our data show a trend towards increased survival with increased time to LVEF recovery, which is counter-intuitive to the prevailing concept that more rapid recovery of LVEF is beneficial. Shorter duration of heart failure is associated with increased likelihood of LVEF recovery, but the association with improved outcomes among HFrecEF has not been shown [22]. Moreover, a post-hoc analysis of the STICH trial demonstrated delayed LVEF recovery (2 years) was associated with improved survival but early normalization (30 days) was not [23].

Patients with HFrecEF have traditionally been considered as a homogenous group. Perhaps not surprisingly, we found mortality in the LVrecEF clusters was associated with concomitant comorbidities that have been associated with worse outcomes in HFpEF patients [24, 25]. For example, patients in clusters 1–2 had the highest creatinine levels, suggesting that underlying chronic kidney disease may have contributed to worsening clinical outcomes. Cluster 1 had the highest mortality, and was comprised of patients with a QRS duration >120ms, the majority of whom did not receive CRT. This cluster had the lowest use of ACEi/ARB and the highest use of loop diuretics, both of which have been associated with poor clinical outcomes [26]. However, we believe that the differences in medication use do not completely account for the high mortality observed in this cluster, insofar as all of the patients who died in Cluster 1 were receiving an ACEi/ARB, and the one patient who survived was not taking an ACEi/ARB. Because the patients for this study were drawn from an administrative data base, we do not know whether the wide QRS was secondary to a RBBB or LBBB, nor do we know the reason why these patients did not receive a CRT. While QRS prolongation secondary to an intraventricular conduction delay that causes mechanical LV dyssynchrony is widely accepted as a mechanism for worsening HF and sudden cardiac death, it is worth noting that the magnitude of benefit obtained from CRT is not uniform across all patterns of QRS prolongation, and CRT does not correlate with hemodynamic and clinical improvement in ~ 30% of patients. Although speculative, the prolonged QRS in patients in Cluster 1 may have identified patients with slow(er) conduction velocity secondary to underlying myopathic disease (e.g. fibrosis, redistribution of connexins, inherited or acquired abnormalities of ion channel abnormalities). Other notable differences in mortality amongst the clusters deserve to be highlighted. Patients in Cluster 7, identified by a high prevalence of ischemic heart disease, had an intermediate risk of mortality. Although the overall prevalence of ischemic heart disease in our cohort was low (~13%), it is consistent with the observation that the majority of patients with ischemic heart disease do not increase their LVEF > 50% on GDMT [27], and hence would not have been included based on the way that this study was designed.

### Limitations

Several limitations to our study warrant discussion. First, this is a retrospective analysis of an administrative data set. Given that patients who present with HFrEF do not always have 2-D echocardiographic follow-up studies that are scheduled at regular intervals, our cohort may not encompass all HFrEF patients who have recovery of their LVEF. Furthermore, the administrative data set does not allow us to determine the total number of HFrEF patients that were screened for recovery of LVEF. Medication data were obtained from the outpatient medical record at Washington University School of Medicine; not all patients captured in this study had follow-up in the affiliated clinics, which limits our ability to adjust the mortality analysis for medication use.

Additionally, we used ICD codes to define co-morbidities which may introduce some error. However, we used a validated method for grouping ICD codes into clinically relevant co-morbidities using the Elixhauser co-morbidity index [9]. We were also unable to include markers of LV size/dimensions given the missingness of data within the 2-D echo data base. Furthermore we did not have accurate follow up information regarding mitral regurgitation. It should be recognized that the clusters found by our unsupervised clustering machine learning method depend on the distribution of our data. Accordingly, the number of clusters and their defining characteristics may have some variance from data set to data set. Our sample size for some clusters is small (e.g. Cluster 1 has 37 patients) which limits precision of the results. Therefore, the results and generalizability of the present study will need to be verified in different patient cohorts, perhaps utilizing a multi-center collaboration to increase sample size.

## Conclusions

In the present study, we used an unsupervised clustering algorithm to identify previously unrecognized clinical phenotypes among patients with LVrecEF. Here we show for the first time that there is significant heterogeneity of clinical phenotypes among LVrecEF patients, and that clustering identifies high and low risk cohorts of LVrecEF patients, which may have important clinical ramifications for developing long term strategies for managing these patients. The optimal clinical management of LVrecEF patients remains challenging because of the dearth of robust prospective data to guide clinical decision making. Relevant to this discussion is the open-label randomized pilot trial, which compared phased withdrawal of GDMT vs continued therapy with GDMT. The TRED-HF trial showed that within 6 months, 44% of the first withdrawal group and 36% of the second group experienced a recurrence of HF, which was defined as a fall in LVEF > 10% to < 50%, an increase in left ventricular end diastolic volume (LVEDV) of > 10% and to higher than the normal range, a doubling of the NTproBNP to > 400 ng/L, or clinical evidence of heart failure [28]. Accordingly, the results of the present study may have direct clinical implications for the management of HF patients with a recovered LVEF, insofar as we were able to use machine learning to identified high risk populations (e.g., Cluster 1) for whom closer surveillance and continued GDMT may be more beneficial, as well as lower risk populations (Clusters 6–7) in whom select withdrawal of GDMT may be possible. These interesting possibilities could be tested in a randomized manner for both high- and low-risk patient populations. Although the results of the present study need to be confirmed in a separate data set, they do suggest it may be possible in the foreseeable future to utilize clustering algorithms to identify LVrecEF patients who are at high risk for recurrent HF events. This can in turn lead to closer clinical follow-up of these patients, as well as facilitate simple, pragmatic clinical trials to determine the optimal GDMT regimen for HFrecEF patients.

## Supporting information

S1 Fig Silhouette width method for determining the optimal method for identifying unique patient clusters. A silhouette analysis suggested that 7 clusters were the optimal number of LVrecEF clusters.
(TIF)

S1 Table Comparison of patients with and without follow up echo. (DOCX)

S1 Data Clean sheet for all patients. (CSV)

S2 Data Echo data. (XLSX)

S3 Data Mortality. (XLSX)

## Abbreviations

ACEi: angiotensin converting enzyme inhibitor
ARB: angiotensin receptor blocker
CRT: cardiac resynchronization therapy
GDMT: guideline directed medical therapy
HF: heart failure
HFpEF: heart failure with preserved ejection fraction
HFrEF: heart failure with reduced ejection fraction
ICD: International Statistical Classification of Diseases and Related Health Problems
LVEF: left ventricular ejection fraction
MRA: mineralocorticoid receptor antagonist

## References

[1] Wilcox JE, Fang JC, Margulies KB, Mann DL. Heart Failure With Recovered Left Ventricular Ejection Fraction: JACC Scientific Expert Panel. J Am Coll Cardiol. 2020;76(6):719–34. 10.1016/j.jacc.2020.05.075 32762907

[2] Wilcox JE, Yancy CW. Heart Failure-A New Phenotype Emerges. JAMA Cardiol. 2016;1(5):507–9. 10.1001/jamacardio.2016.1356 27434185

[3] Basuray A, French B, Ky B, Vorovich E, Olt C, Sweitzer NK, et al. Heart failure with recovered ejection fraction: clinical description, biomarkers, and outcomes. Circulation. 2014;129(23):2380–7. 10.1161/CIRCULATIONAHA.113.006855 24799515 PMC4053508

[4] Merlo M, Stolfo D, Anzini M, Negri F, Pinamonti B, Barbati G, et al. Persistent recovery of normal left ventricular function and dimension in idiopathic dilated cardiomyopathy during long-term follow-up: does real healing exist? J Am Heart Assoc. 2015;4(1):e001504. 10.1161/JAHA.114.000570 25587018 PMC4330074

[5] Mann DL, Barger PM, Burkhoff D. Myocardial recovery: myth, magic or molecular target? J Amer Coll Cardiol. 2012;60:2465–72 10.1016/j.jacc.2012.06.062 23158527 PMC3522780

[6] Deo RC. Machine Learning in Medicine. Circulation. 2015;132(20):1920–30. 10.1161/CIRCULATIONAHA.115.001593 26572668 PMC5831252

[7] Shah SJ, Katz DH, Selvaraj S, Burke MA, Yancy CW, Gheorghiade M, et al. Phenomapping for novel classification of heart failure with preserved ejection fraction. Circulation. 2015;131(3):269–79. 10.1161/CIRCULATIONAHA.114.010637 25398313 PMC4302027

[8] Thavendiranathan P, Grant AD, Negishi T, Plana JC, Popović ZB, Marwick TH. Reproducibility of echocardiographic techniques for sequential assessment of left ventricular ejection fraction and volumes: application to patients undergoing cancer chemotherapy. J Am Coll Cardiol. 2013;61(1):77–84. 10.1016/j.jacc.2012.09.035 23199515

[9] Elixhauser A, Steiner C, Harris DR, Coffey RM. Comorbidity measures for use with administrative data. Med Care. 1998;36(1):8–27. 10.1097/00005650-199801000-00004 9431328

[10] Florea VG, Rector TS, Anand IS, Cohn JN. Heart Failure With Improved Ejection Fraction: Clinical Characteristics, Correlates of Recovery, and Survival: Results From the Valsartan Heart Failure Trial. Circ Heart Fail. 2016;9(7). 10.1161/CIRCHEARTFAILURE.116.003123 27413037

[11] Park CS, Park JJ, Mebazaa A, Oh IY, Park HA, Cho HJ, et al. Characteristics, Outcomes, and Treatment of Heart Failure With Improved Ejection Fraction. J Am Heart Assoc. 2019;8(6):e011077. 10.1161/JAHA.118.011077 30845873 PMC6475046

[12] Kashani A, Barold SS. Significance of QRS complex duration in patients with heart failure. J Am Coll Cardiol. 2005;46(12):2183–92. 10.1016/j.jacc.2005.01.071 16360044

[13] Zafrir B, Lund LH, Laroche C, Ruschitzka F, Crespo-Leiro MG, Coats AJS, et al. Prognostic implications of atrial fibrillation in heart failure with reduced, mid-range, and preserved ejection fraction: a report from 14 964 patients in the European Society of Cardiology Heart Failure Long-Term Registry. Eur Heart J. 2018;39(48):4277–84. 10.1093/eurheartj/ehy626 30325423

[14] Kutyifa V, Kloppe A, Zareba W, Solomon SD, McNitt S, Polonsky S, et al. The influence of left ventricular ejection fraction on the effectiveness of cardiac resynchronization therapy: MADIT-CRT (Multicenter Automatic Defibrillator Implantation Trial With Cardiac Resynchronization Therapy). J Am Coll Cardiol. 2013;61(9):936–44. 10.1016/j.jacc.2012.11.051 23449428

[15] Swat SA, Cohen D, Shah SJ, Lloyd-Jones DM, Baldridge AS, Freed BH, et al. Baseline Longitudinal Strain Predicts Recovery of Left Ventricular Ejection Fraction in Hospitalized Patients With Nonischemic Cardiomyopathy. J Am Heart Assoc. 2018;7(20):e09841. 10.1161/JAHA.118.009841 30371257 PMC6474980

[16] Ruwald MH, Solomon SD, Foster E, Kutyifa V, Ruwald AC, Sherazi S, et al. Left ventricular ejection fraction normalization in cardiac resynchronization therapy and risk of ventricular arrhythmias and clinical outcomes: results from the Multicenter Automatic Defibrillator Implantation Trial With Cardiac Resynchronization Therapy (MADIT-CRT) trial. Circulation. 2014;130(25):2278–86. 10.1161/CIRCULATIONAHA.114.011283 25301831

[17] Stolfo D, Merlo M, Pinamonti B, Poli S, Gigli M, Barbati G, et al. Early improvement of functional mitral regurgitation in patients with idiopathic dilated cardiomyopathy. Am J Cardiol. 2015;115(8):1137–43. 10.1016/j.amjcard.2015.01.549 25721482

[18] Hellawell JL, Margulies KB. Myocardial Reverse Remodeling. Cardiovasc Ther. 2012;20:172–81. 10.1111/j.1755-5922.2010.00247.x 21108773

[19] Givertz MM, Mann DL. Epidemiology and Natural History of Recovery of Left Ventricular Function in Recent Onset Dilated Cardiomyopathies. Curr Heart Fail Rep. 2013;10 321–30. 10.1007/s11897-013-0157-5 24014141 PMC3823811

[20] Adamo L, Perry A, Novak E, Makan M, Lindman BR, Mann DL. Abnormal Global Longitudinal Strain Predicts Future Deterioration of Left Ventricular Function in Heart Failure Patients With a Recovered Left Ventricular Ejection Fraction. Circ Heart Fail. 2017;10(6). 10.1161/CIRCHEARTFAILURE.116.003788 28559418 PMC5505492

[21] Lupon J, Diez-Lopez C, de Antonio M, Domingo M, Zamora E, Moliner P, et al. Recovered heart failure with reduced ejection fraction and outcomes: a prospective study. Eur J Heart Fail. 2017;19(12):1615–23. 10.1002/ejhf.824 28387002

[22] Cioffi G, Stefenelli C, Tarantini L, Opasich C. Chronic left ventricular failure in the community: Prevalence, prognosis, and predictors of the complete clinical recovery with return of cardiac size and function to normal in patients undergoing optimal therapy. J Card Fail. 2004;10(3):250–7. 10.1016/j.cardfail.2003.10.002 15190536

[23] In Ischemic Cardiomyopathy, Delayed But Not Early Normalization of The Ejection Fraction Is Associated With Reduced Mortality. Journal of Cardiac Failure. 2020;26(10, Supplement):S48.

[24] Ather S, Chan W, Bozkurt B, Aguilar D, Ramasubbu K, Zachariah AA, et al. Impact of noncardiac comorbidities on morbidity and mortality in a predominantly male population with heart failure and preserved versus reduced ejection fraction. J Am Coll Cardiol. 2012;59(11):998–1005. 10.1016/j.jacc.2011.11.040 22402071 PMC4687406

[25] Paulus WJ, Tschöpe C. A Novel Paradigm for Heart Failure With Preserved Ejection Fraction. Comorbidities Drive Myocardial Dysfunction and Remodeling Through Coronary Microvascular Endothelial Inflammation. 2013;62(4):263–71. 10.1016/j.jacc.2013.02.092 23684677

[26] Levy WC, Mozaffarian D, Linker DT, Sutradhar SC, Anker SD, Cropp AB, et al. The Seattle Heart Failure Model: prediction of survival in heart failure. Circulation. 2006;113(11):1424–33. 10.1161/CIRCULATIONAHA.105.584102 16534009

[27] Lupon J, Gavidia-Bovadilla G, Ferrer E, de Antonio M, Perera-Lluna A, Lopez-Ayerbe J, et al. Dynamic Trajectories of Left Ventricular Ejection Fraction in Heart Failure. J Am Coll Cardiol. 2018;72(6):591–601. 10.1016/j.jacc.2018.05.042 30071987

[28] Halliday BP, Wassall R, Lota AS, Khalique Z, Gregson J, Newsome S, et al. Withdrawal of pharmacological treatment for heart failure in patients with recovered dilated cardiomyopathy (TRED-HF): an open-label, pilot, randomised trial. Lancet. 2019;393:61–73. 10.1016/S0140-6736(18)32484-X 30429050 PMC6319251

