# Supplementary figures and images for: Unsupervised cluster analysis of patients with recovered left ventricular ejection fraction identifies unique clinical phenotypes
Source: PLoS One. 2021 Mar 18;16(3):e0248317. doi: 10.1371/journal.pone.0248317 (PMC7971566; doi:10.1371/journal.pone.0248317)

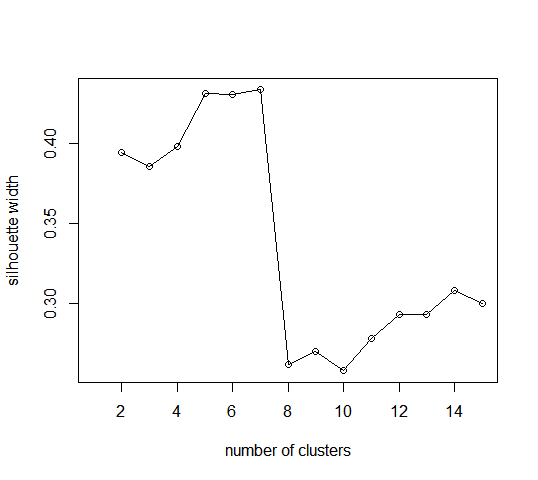

Supplement: S1 Fig — A silhouette analysis suggested that 7 clusters were the optimal number of LVrecEF clusters. (TIF) [file pone.0248317.s001.tif]
