# Supplementary material for: Unsupervised cluster analysis of patients with recovered left ventricular ejection fraction identifies unique clinical phenotypes
Source: PLoS One. 2021 Mar 18;16(3):e0248317. doi: 10.1371/journal.pone.0248317 (PMC7971566; doi:10.1371/journal.pone.0248317)
Supplement: S1 Table — (DOCX) [file pone.0248317.s002.docx]

| **S1 Table: Comparison of Patients with and without follow up echo** | | | |
| --- | --- | --- | --- |
|  | **Follow-up echo** | **No Follow-up echo** | **p-value** |
| **n** | 488 | 401 |  |
| **Age (median [IQR])** | 61 [51, 70] | 62 [52, 72] | 0.108 |
| **BMI (median [IQR])** | 24.2 [20.1, 28.5] | 23.8 [20.5, 27.9] | 0.735 |
| **Female (%)** | 218 (44.8) | 197 (49.1) | 0.219 |
| **Minority (%)** | 180 (36.9) | 139 (34.7) | 0.537 |
| **Systolic blood pressure (median [IQR])** | 123 [106, 139] | 121 [106, 139] | 0.804 |
| **Atrial fibrillation (%)** | 142 (29.1) | 64 (16.0) | <0.001 |
| **Diabetes (%)** | 87 (17.8) | 44 (11.0) | 0.006 |
| **Hemoglobin mg/dL (median [IQR])** | 11.2 [9.5, 13.0] | 10.7 [9.4, 12.6] | 0.033 |
| **Sodium mmol/L (median [IQR])** | 139 [137, 142] | 140 [137, 142] | 0.219 |
| **Creatinine (median [IQR])** | 1.17 [0.88, 1.64] | 1.02 [0.76, 1.60] | 0.017 |
| **Ischemic heart disease (%)** | 84 (17.2) | 35 (8.7) | <0.001 |
| **Cardiac resynchronization therapy (%)** | 139 (28.5) | 33 (8.2) | <0.001 |
| **Time to recovery, days (median [IQR])** | 661 [238, 1368] | 259 [41, 845] | <0.001 |
| **Pre-recovery EF (median [IQR])** | 26 [20, 30] | 29 [23, 33] | <0.001 |
| **Recovered EF (median [IQR])** | 59 [54, 63] | 58 [54, 63] | 0.264 |
| **Change in EF (median [IQR])** | 29 [22, 37] | 29 [23, 36] | 0.694 |
| **Mod-Severe MR (%)** | 29 (5.9) | 20 (5.0) | 0.636 |
| **QRS≥120ms (%)** | 25 (5.1) | 24 (6.0) | 0.68 |
| **ACE/ARB (%)** | 201 (57.4) | 119 (53.6) | 0.417 |
| **Beta-blocker (%)** | 238 (68.0) | 152 (68.5) | 0.98 |
| **MRA (%)** | 87 (24.9) | 50 (22.5) | 0.591 |
| **Loop diuretic (%)** | 162 (46.3) | 76 (34.2) | 0.006 |
